# Mononucleotide repeat expansions with non-natural polymerase substrates

**DOI:** 10.1038/s41598-021-82150-2

**Published:** 2021-01-28

**Authors:** Alexander V. Chudinov, Vadim A. Vasiliskov, Viktoriya E. Kuznetsova, Sergey A. Lapa, Natalia A. Kolganova, Edward N. Timofeev

**Affiliations:** grid.418899.5 0000 0004 0619 5259 W. A. Engelhardt Institute of Molecular Biology Russian Academy of Sciences, Vavilov St. 32, Moscow, Russia 119991

**Keywords:** Biochemistry, Chemical biology

## Abstract

Replicative strand slippage is a biological phenomenon, ubiquitous among different organisms. However, slippage events are also relevant to non-natural replication models utilizing synthetic polymerase substrates. Strand slippage may notably affect the outcome of the primer extension reaction with repetitive templates in the presence of non-natural nucleoside triphosphates. In the current paper, we studied the ability of Taq, Vent (exo-), and Deep Vent (exo-) polymerases to produce truncated, full size, or expanded modified strands utilizing non-natural 2′-deoxyuridine nucleotide analogues and different variants of the homopolymer template. Our data suggest that the slippage of the primer strand is dependent on the duplex fluttering, incorporation efficiency for a particular polymerase-dNTP pair, rate of non-templated base addition, and presence of competing nucleotides.

## Introduction

Modified deoxynucleoside triphosphates (dNTPs) have found wide application in the synthesis of modified DNA and particularly in the generation of high-affinity DNA aptamers by numerous SELEX methods^1^. At present, the majority of SELEX techniques^2^, including cell-SELEX, CE-SELEX, or microfluidic-based SELEX, may be easily adapted for the use of non-natural nucleotide substrates^3,4^. The main advantage that modified nucleotides bring to the process of directed evolution is the notably expanded chemical diversity of oligonucleotide libraries^5^. This parameter appeared to be critical for the selection of an aptamer with high affinity and specificity. Additionally, chemical modifications usually result in an increased resistance to biodegradation. One of the major characteristics of non-natural dNTPs is their substrate properties with respect to DNA polymerases. The estimation of this parameter in the primer extension (PEX) reaction is a routine procedure in the characterization of newly synthesized modified dNTPs^6–9^. In particular, homopolymer DNA templates may be used for the estimation of the polymerization efficiency of non-natural nucleotides to verify the possibility of multiple consecutive incorporations^10,11^. Recently, we described the slippage effect in a modified primer strand that may result in overestimation of the substrate characteristics of non-natural dNTPs^12^. Slippage is a well-known phenomenon that is largely associated with microsatellite DNA comprising short repetitive sequences^13^. The formation of misaligned dsDNA with bulged or looped sites results in enzymatic slippage, which accounts for short indel mutations^14^.

**Figure 1:**
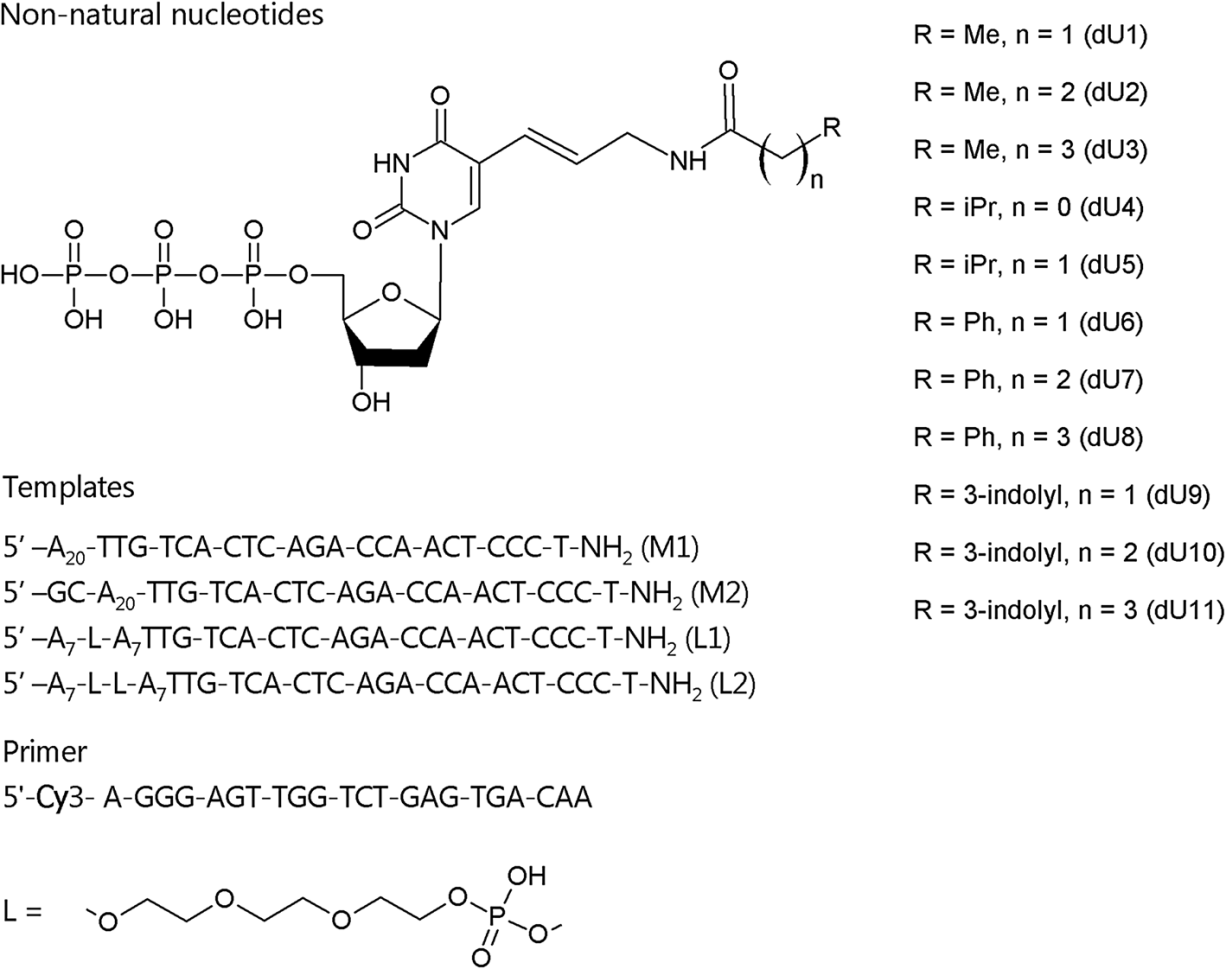
The structure of modified 2′-deoxyuridine-5′-triphosphates and sequences of DNA templates and the labelled primer.

In this paper, we describe the slippage effect that was observed when using new dUTP analogues with non-natural side chains at C5 of the pyrimidine heterocycle^15,16^ (Fig. 1). PEX reactions over a few variants of the homopolymer template in the presence of Taq, Vent (exo-), and Deep Vent (exo-) DNA polymerases revealed the important role of non-templated base addition, presence of other nucleotide substrates, and polymerase efficiency with respect to the selected dNTP analogue in generating slippage products. We also demonstrated that the slippage mechanism may mimic polymerase jumps over non-natural template inserts.

## Results and discussion

**Figure 2:**
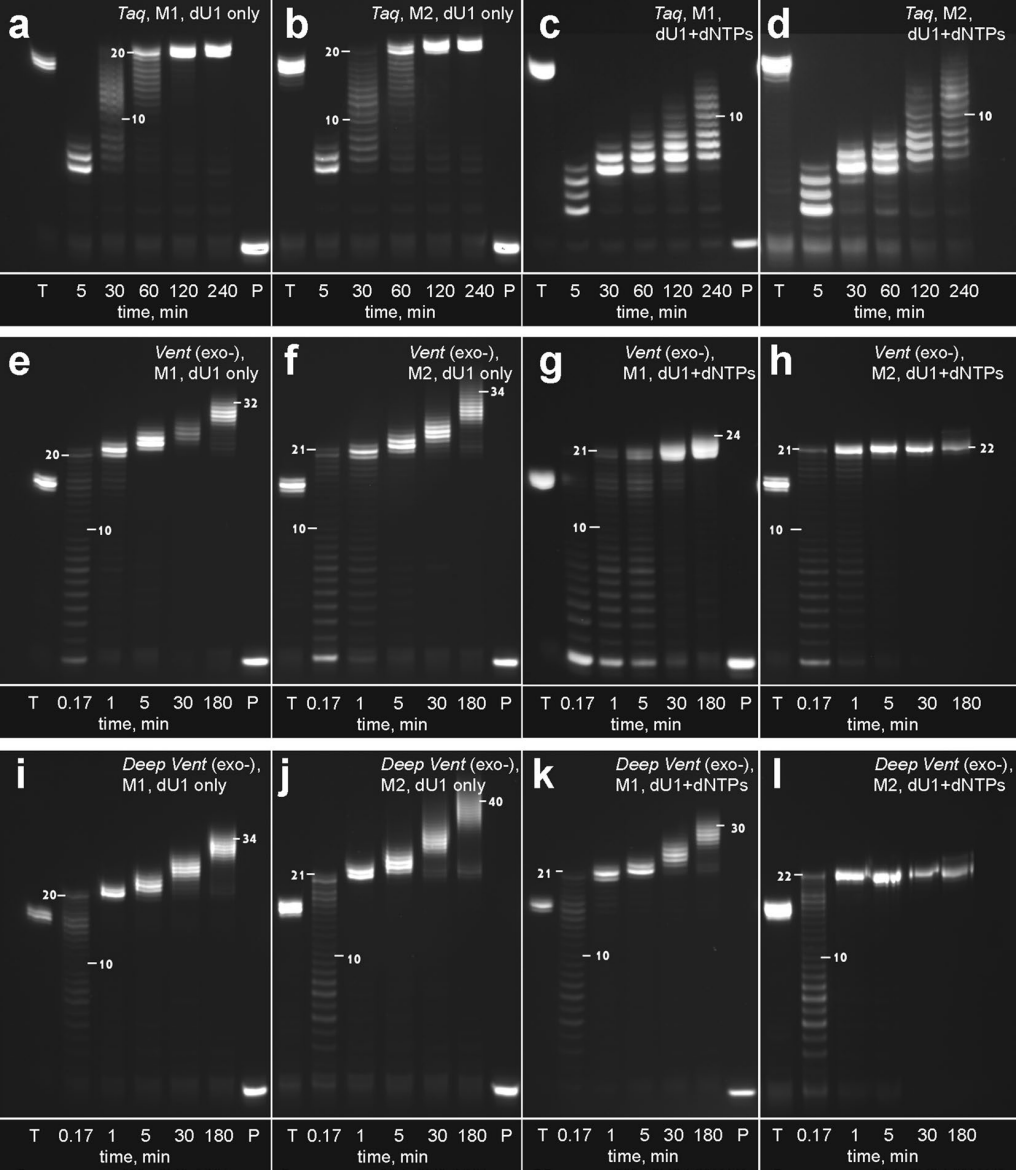
Electrophoretic separation of the labelled extension products formed in the PEX reaction with dU1 and templates M1/M2 in the presence of Taq (**a**–**d**), Vent (exo-) (**e**–**h**), and Deep Vent (exo-) (**i**–**l**) DNA polymerases. Lane T: TTP/Taq polymerase, 1 h. Lane P: primer. The abbreviation “dNTPs” here means a mixture of dATP, dCTP and dGTP. The numbers indicate the difference in the length between the selected fragment and the primer. Source gel images are presented in Supplementary Figure S5.

Recently reported modified dUTP analogues dU1–dU11^15,16^ were studied as nucleotide substrates in the PEX reaction with Taq, Vent (exo-), and Deep Vent (exo-) DNA polymerases, enzymes commonly used in SELEX applications. The latter two polymerases are known to be quite efficient in extending the primer strand with non-natural nucleotide substrates^1,5^. Particularly, Vent (exo-) DNA polymerase is one of the most frequently used commercially available enzymes when studying modified nucleoside triphosphates. The modified nucleotide dU1 was selected for a detailed study of the slippage effect. Homopolymer sequence dA_20_ beyond the primer region was used in templates M1 and M2 (Fig. 1). Two variants of the template strand contained either a 5′-dA_20_-3′ (M1) or 5′-GCA_20_-3′ (M2) extension site. Templates were protected from enzymatic transformation of their 3′ ends by adding an amino group linker. To estimate the role of the GC cap and natural nucleotides in suppressing the slippage, we ran the extension with dU1 only or in a mixture with the other three natural dNTPs. The results of PEX are shown in Fig. 2. In the presence of Taq polymerase and dU1, the PEX reaction yielded the full size modified strand containing a one or two nucleotide overhang after 1 h of exposure at 72 °C (Fig. 2a,b). Adding a dATP, dCTP, and dGTP nucleotide mixture to the reaction was detrimental for generating the full-size modified product. We could only detect 5–8 added residues of dU1 after 1 h of the reaction (Fig. 2c,d).

The PEX reaction in the presence of Vent (exo-) DNA polymerase strikingly differed from that with Taq polymerase in the time required to generate the full-size modified strand. In the case of Vent (exo-) polymerase, the time scale for the extension by twenty modified residues was within the interval of 1–5 min. Additionally, Vent (exo-) polymerase induced a notable slippage effect. In the absence of natural nucleotides, the length of the modified strand after 3 h of exposure was in the range of 30–40 nt beyond the primer site (Fig. 2e,f). Adding dATP, dCTP, and dGTP to the PEX reaction with uncapped template reduced the maximal product length (Fig. 2g). Nevertheless, we could still observe products with up to 24 added non-natural nucleotides. Only the combination of the 5′ GC-capped template and added natural nucleotides inhibited slippage of the primer strand (Fig. 2h). Nevertheless, even in this case, the final product contained a 1–2 nt overhang. The PEX reaction with Deep Vent (exo-) DNA polymerase induced a product distribution similar to that observed with Vent (exo-) (Fig. 2i–l). However, in this case, the slippage effect was more pronounced. Extension over the GC-capped template in the presence of a single nucleotide substrate dU1 yielded the highest length of the modified strand. The most efficient suppression of slippage was achieved by using the combination of a capped template and a mixture of nucleotides (Fig. 2h,l).

A study of other C5-modified dUTPs with the template M1 and three polymerases revealed that slippage of the primer strand is a typical outcome of the PEX reaction over a homopolymer template (Table 1). We observed similar behaviours for other modified dUTPs except those with an extremely low efficiency of incorporation. Since the slippage effect is not specific for modified nucleotides, it was also observed for natural dTTP and dATP, as shown in Figures S1 and S2. Polymerases Vent (exo-) and Deep Vent (exo-) were able to extend the poly(dT) strand up to 30 nt in the presence of other natural nucleotides. Although we observed some difference between Taq DNA polymerase and two other polymerases in promoting slippage with natural dTTP (Fig. S1), longer exposure with Taq polymerase similarly results in the formation of a slipped primer strand. In the case of dATP, the slippage effect was even more pronounced, yielding long PEX products with more than 40 added nucleotides (Figure S2). We associate the formation of longer products by natural dATP with a higher contribution of non-template base addition to the overall slippage mechanism, as discussed below.

**Table 1:** Results of the PEX reaction with modified nucleotide substrates.

dU analog	dU1	dU2	dU3	dU4	dU5	dU6	dU7	dU8	dU9	dU10	dU11
**M1 + dU variant**											
Polymerase	Taq										
N_max_^a^	22	20	14	5	6	13	13	11	11	11	12
*t*, min^b^	30	240	240	240	30	240	30	30	240	30	30
Polymerase	Vent (exo-)										
N_max_	34	28	25	29	26	21	19	16	16	15	13
*t*, min	5	5	30	5	5	5	5	5	5	5	5
Polymerase	Deep Vent (exo-)										
N_max_	36	32	32	31	27	23	20	16	20	17	14
*t*, min	5	5	5	5	5	5	5	5	5	5	5

dU analog	dU1	dU2	dU3	dU4	dU5	dU6	dU7	dU8	dU9	dU10	dU11
**M1 + dU variant + d(AGC)**											
Polymerase	Taq										
N_max_	14	6	6	5	5	5	8	8	7	11	10
*t*, min	240	240	240	120	30	30	30	30	30	30	30
Polymerase	Vent (exo-)										
N_max_	24	23	21	23	22	21	17	15	15	15	12
*t*, min	5	5	30	5	5	5	5	5	5	5	5
Polymerase	Deep Vent (exo-)										
N_max_	32	28	25	26	25	23	19	15	20	16	13
*t*, min	5	5	5	5	5	5	5	5	5	5	5

^a^The maximum number of added residues after 4 h (Taq polymerase) or 3 h (Vent (exo-) and Deep Vent (exo-) polymerases) of the PEX reaction.

^b^The timescale of the standard PEX experiment that yielded products with ≥ 20 added residues or produced the most final products observed at the maximum exposure time.

Much faster formation of the full-size modified strand in the presence of Vent (exo-) and Deep Vent (exo-) DNA polymerases confirmed the fact that Taq polymerase incorporates modified nucleotides inefficiently. However, the ability of Taq polymerase to build full length product over time and the clear effect of the added mixture of dATP, dCTP, and dGTP suggests a notable contribution of non-templated synthesis. The transferase activity of Taq polymerase at the ends of misaligned duplexes is supposed to be a major driver of extension and the formation of the full-length product. In the presence of the nucleotide mixture, purine nucleotides successfully compete with dU1 for non-templated terminal incorporation, thus blocking the slippage. To support non-templated base addition, poly-dA template is expected to form bulges that allow close alignment of the 3′ end of the primer strand and 5′ end of the template. This mechanism implies a certain frequency of nucleation/dissociation events in a misaligned DNA duplex^17^ and specific ratio between the rates of templated and non-templated synthesis. Additionally, the pausing and dissociation of polymerase from the ternary complex is required for slippage to proceed, as have been proposed in the literature^18^.

The higher efficiency of incorporation in the cases of Vent (exo-) and Deep Vent (exo-) allows quick extension with dU1. Only a minor blocking effect on the slippage was observed for the addition of the nucleotide mixture in PEX with uncapped template. This result points to a much lower contribution of transferase activity in the extension with Vent (exo-) and Deep Vent (exo-) polymerases. The formation of an overextended modified product in the reaction with these two polymerases implies a template-guided extension of misaligned duplexes. Unlike the non-templated base addition, this scenario requires a bulge formation in the primer strand. However, this is not the case for the GC-capped template in the presence of the nucleotide mixture since quick extension prevents the formation of misaligned duplexes suitable for further templated or non-templated synthesis.

It is noteworthy that fluttering of the duplex is one of the major driving factors of the slippage effect. It has been shown that dynamics of the terminal bases is responsible for slippage and indel mutations near DNA termini^19–21^. The nucleation-zipping mechanism of DNA duplex formation^17,22^ suggests that the formation of misaligned duplexes at mononucleotide repetitive templates is a very likely event at temperatures close to T_m_. We measured the thermal stability of the full length DNA duplex with either a natural or modified primer strand. Melting of the duplex was monitored by FRET between Cy5 at the 3′ end of the template and Cy3 at the 5′ end of the primer strand (Fig. S3). The T_m_ values for native and modified duplexes were approximately 70 °C, which is very close to the selected conditions of PEX. Apparently, extension at 72 °C favours random nucleation between poly-dA and the respective complementary strand.

**Figure 3:**
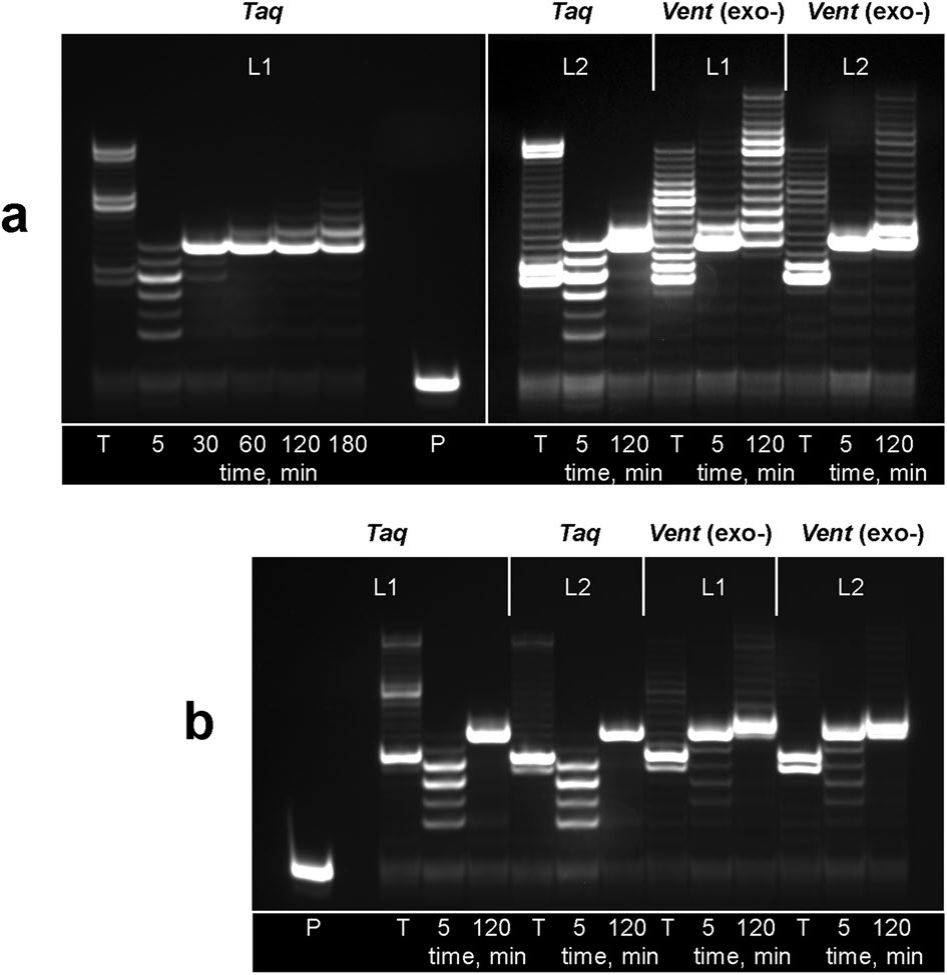
Electrophoretic separation of the labelled extension products formed in the PEX reaction with TTP/dU1 and templates L1/L2 in the absence (**a**) or presence (**b**) of the dATP, dCTP, and dGTP mixture. Lane T: TTP, 1 h. Lane P: primer. Lanes with time labels: dU1. Source gel images are presented in Supplementary Figure S5.

Duplex flapping and an ability to form slipped structures suggests that, at least for homopolymer sequences, it is not mandatory to retain uninterrupted template to support the enzymatic synthesis of the primer strand. In the case that the template contains poly-dA tracts, it presumably may be extended in the presence of dTTP or modified dUTPs regardless of the presence of other sequence context. To verify an ability of polymerase to extend the template with an interrupted poly-dA motif, we designed two template strands (L1 and L2) that consisted of two dA_7_ tracts connected by a single or double triethyleneglycol spacer (Fig. 1). In addition to sequence modification, the presence of a non-extendable synthetic linker in the template was expected to increase the frequency of flapping. The results of the PEX reaction in the presence of Taq and Vent (exo-) DNA polymerases are shown in Fig. 3. Taq polymerase was able to extend the chimeric template L1 up to 20 nt for 2 h when using natural dTTP. Interestingly, it is seen from the gel image that the polymerase was paused three times. This observation suggests that the dA_7_ tract of the composite template L1 was extended in triplicate, while further cycles were restricted. Modified nucleotide dU1 was incorporated only along a single dA_7_ tract after 30 min of exposure. Further elongation was slow, and the highest extension was detectable at 11 added residues after 4 h of exposure. Extension with dTTP over the template L2 in the presence of Taq polymerase also showed the longest primer strand with 20 added nucleotides. However, in this case, only two well-defined paused points were detected. Expectedly, in the case of the L2 template, a major band for dU1 was also observed at 7 added residues with Taq polymerase. The efficiency of slippage was notably increased for the modified nucleotides in the presence of Vent (exo-) DNA polymerase. Both L1 and L2 templates were extended with modified residues up to 21 added nucleotides. The PEX reaction with the template L1 clearly showed a three-segment pattern for dTTP and dU1 in this case. The slipped strand was formed by dTTP with lower efficiency in the presence of Vent (exo-) polymerase compared to PEX with Taq polymerase. The extension of the template L2 was even less efficient, but the longest primer strands were still detectable for both dTTP and dU1. Predictably, adding the mixture of nucleotides dATP, dCTP, and dGTP considerably reduced the yield of slipped strands (Fig. 3b).

To verify the role of duplex fluttering in the slippage with non-natural template, we carried out PEX in the presence of Taq DNA polymerase with the artificial template containing a triethyleneglycol insert and thymidine as the nucleotide substrate at two different temperatures, 64 and 72 °C. A higher proportion of the duplex fraction at 64 °C is expected to correlate with the lower frequency of fluttering. As a result, substantially reduced slippage efficiency was observed at this temperature, showing only two extended segments. Increasing the temperature to 72 °C allowed the addition of a third extended dT-tract (Fig. S4). Although, we consider the flapping frequency and dissociation/association events as major drivers of the slippage effect, the observed temperature dependence may be partially attributed to the lower transferase activity of Taq polymerase at 64 °C.

The results of the PEX reaction with chimeric templates prove that the extension of homopolymer templates is supported by the slippage mechanism even in the presence of synthetic inserts. The configuration of the final extension product should most likely be attributed to the duplex scaffold containing triethyleneglycol and (dT)_6_ or (dU1)_6–7_ bulges in the template and the primer strand, respectively. This structure seems to be stable enough to restrict further slippage of the primer strand. Alternatively, the triplex structure may be suggested for the final product with the third segment of the primer strand folded back to form Hoogsteen base pairing^23^. Overall, our data suggest that overextension is a result of interplay between numerous parameters, among which the frequency of duplex fluttering, incorporation efficiency for particular polymerase-dNTP pairs, rate of non-templated base addition, and presence of competing nucleotide substrates seem to be the most important. Indeed, the fluttering of a homopolymer duplex generates a wide range of misaligned strand combinations that are used to further incorporate nucleotides, either in a template-guided mode or through non-templated base addition. The ratio between the rates of these processes determines the contribution of each pathway to overextension. Finally, the presence of the nucleotide mixture is responsible for termination of slippage through non-templated competitive addition of non-homologous base at the 3′ end of the primer strand. With regards to practical aspects, our results demonstrate that the slippage effect may be inhibited by the proper design of the template and using a mixture of dNTPs when studying the substrate properties of modified nucleotides even with a homopolymer template. In addition, the previously described 5′-modification of the template strand may be used to inhibit the non-templated base addition^24^. Importantly, studies of non-natural composite templates point to the possibilities of using slippage-driven extension for controlled enzymatic synthesis over artificial DNA chimaeras.

## Materials and methods

The primer and templates were synthesized using an ABI 3400 DNA/RNA synthesizer and purified by reverse phase HPLC. Triethylene glycol phosphoramidite, amino-linker phosphoramidite, and amino-linker CPG were purchased from Glen Research. Modified dUTPs were prepared by the procedures reported previously^15,16^. Primer labelling was performed using activated Cy3 dye (Lumiprobe). DNA polymerases were purchased from New England Biolabs and SybEnzyme.

### PEX reactions

The reaction mixture (25 μL) contained 4 μM primer and template. The dNTP concentrations were 200 μM (each) in corresponding buffer. ThermoPol® buffer (New England Biolabs) was used for the Vent (exo-) and Deep Vent (exo-) polymerases. A buffer supplied by the manufacturer was used in the reactions with Taq polymerase. Five units of enzyme were used for all polymerases. The reaction mixtures were heated to 95 °C for 1 min and subsequently cooled to 55 °C for 1 min. PEX reactions were performed at 72 °C (if not specified) for varying amounts of time. The reaction was stopped by precipitation with 2% LiClO_4_ in acetone.

### Electrophoresis and detection

Strand separation after the PEX reactions was performed on a 20% polyacrylamide (19:1) gel containing 1xTBE and 7 M urea. Electrophoresis was performed using 1xTBE as the running buffer at 60 °C and 30 V/cm. PEX reaction mixtures were precipitated with 2% LiClO_4_ in acetone and diluted in 10 μL of 7 M urea in D_2_O. Before loading onto the gel, the samples were heated for 1 min at 95 °C. The fluorescent bands were visualized in Cy3 channels using a research custom-made imaging system.

### FRET melting of duplexes

Labelled M1 strand was prepared by treatment of the M1 template with activated Cy5 dye. Complementary Cy3 labelled strands containing thymidines or dU1 residues were synthesized enzymatically at 72 °C with Taq DNA polymerase for 1 h and 2 h, respectively, as described for the PEX reactions. The purification of PEX products was carried out using electrophoresis in 20% denaturing polyacrylamide gel containing 7 M urea. Complementary strands were annealed at a 5 µM concentration in 25 mM TrisHCl (pH 7.5) and 50 mM NaCl. FRET melting was performed with a Cary Eclipse fluorescence spectrophotometer (Agilent Technologies) equipped with a Peltier cell holder. The sample volume was 30 µL. Mineral oil was used to prevent sample evaporation. The heating rate was 0.5 °C/min. The excitation and emission wavelengths were 550 and 663 nm, respectively. The melting points were determined from derivative plots of the melting curves.

## Supplementary Information

Supplementary Information.
