# Supplementary material for: Mononucleotide repeat expansions with non-natural polymerase substrates
Source: Sci Rep. 2021 Jan 28;11:2423. doi: 10.1038/s41598-021-82150-2 (PMC7844250; doi:10.1038/s41598-021-82150-2)
Supplement: Supplementary file 1 — Supplementary Information. [file 41598_2021_82150_MOESM1_ESM.pdf]

Supplementary information

Mononucleotide repeat expansions with non-natural polymerase substrates

Alexander V. Chudinov, Vadim A. Vasiliskov, Viktoriya E. Kuznetsova, Sergey A. Lapa, Natalia A. Kolganova, Edward N. Timofeev*

W. A. Engelhardt Institute of Molecular Biology Russian Academy of Sciences, Moscow 119991, Russia.

*Corresponding author: Engelhardt Institute of Molecular Biology, Russian Academy of Sciences, Vavilov St. 32, 119991 Moscow, Russia; Phone: +7(499) 135-6591; E-mail: edward@eimb.ru

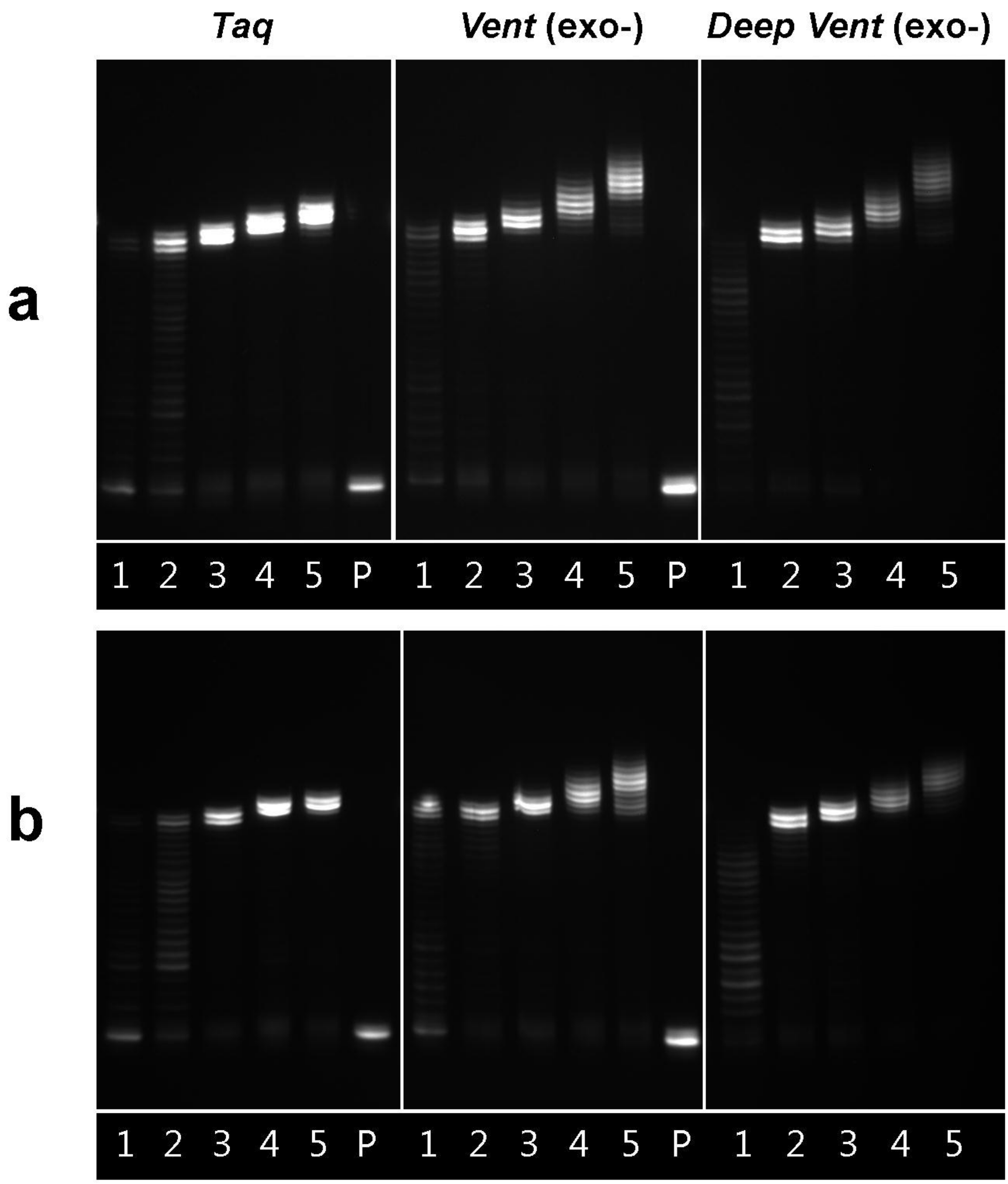

Figure S1. Electrophoretic separation of the labelled extension products formed in the PEX reaction with TTP and template M1 in the absence (a) or presence (b) of the dATP, dCTP, and dGTP mixture. Lanes 1-5: 10 sec, 1 min, 5 min, 30 min, and 3 h. Lane P: primer.

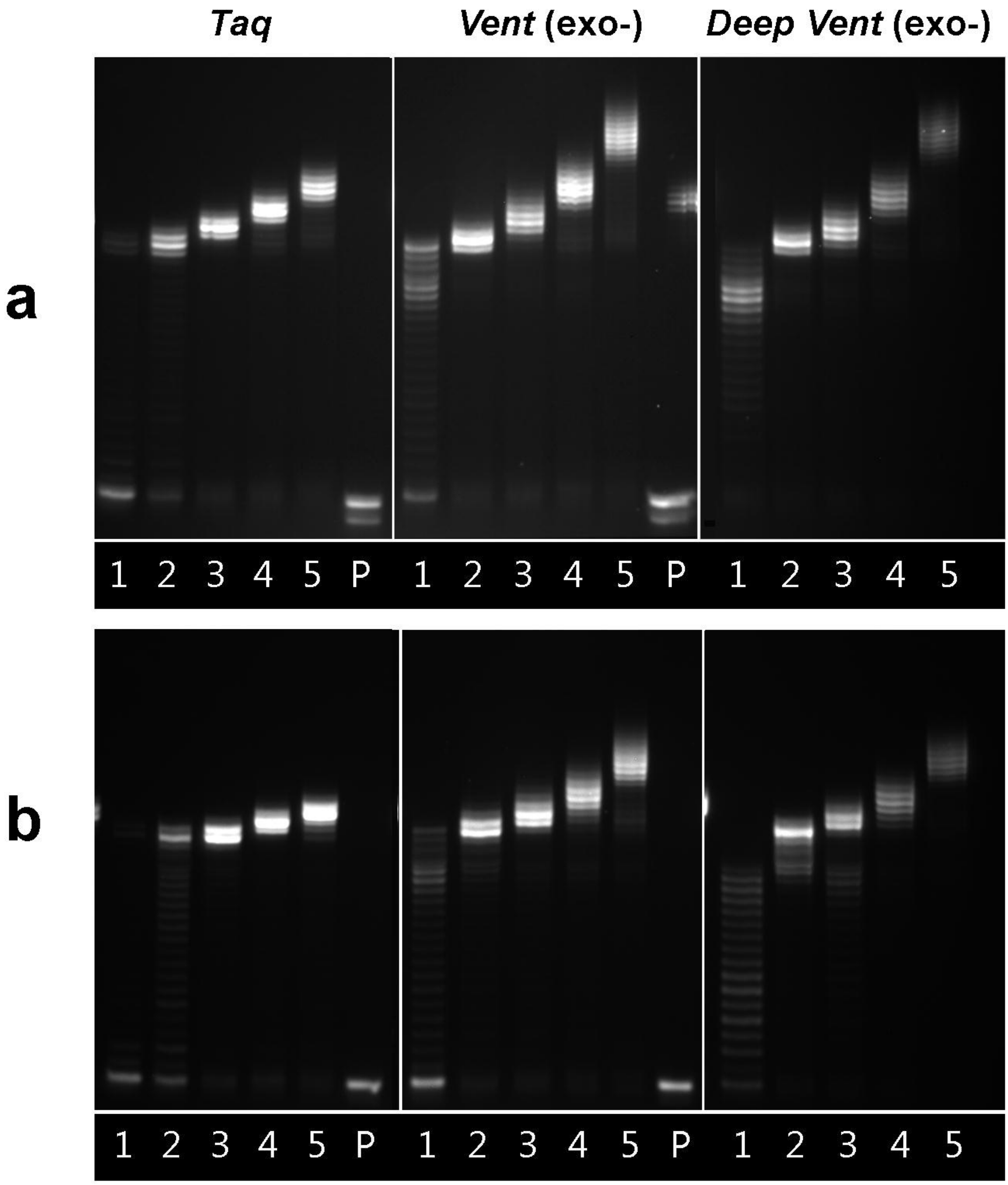

Figure S2. Electrophoretic separation of the labelled extension products formed in the PEX reaction with dATP and template 5'-(T)-TTG-TCA-CTC-AGA-CCA-ACT-CCC-T-NH_2-3' in the absence (a) or presence (b) of the TTP, dCTP, and dGTP mixture. Lanes 1-5: 10 sec, 1 min, 5 min, 30 min, and 3 h. Lane P: primer.

3′-Cy5-TCCCTCAACCAGACTCACTGTT-(A)$_{20}$
5′-Cy3-AGGGAGTTGGTCTGAGTGACAA-(N)$_{20-22}$ N = T or dU*

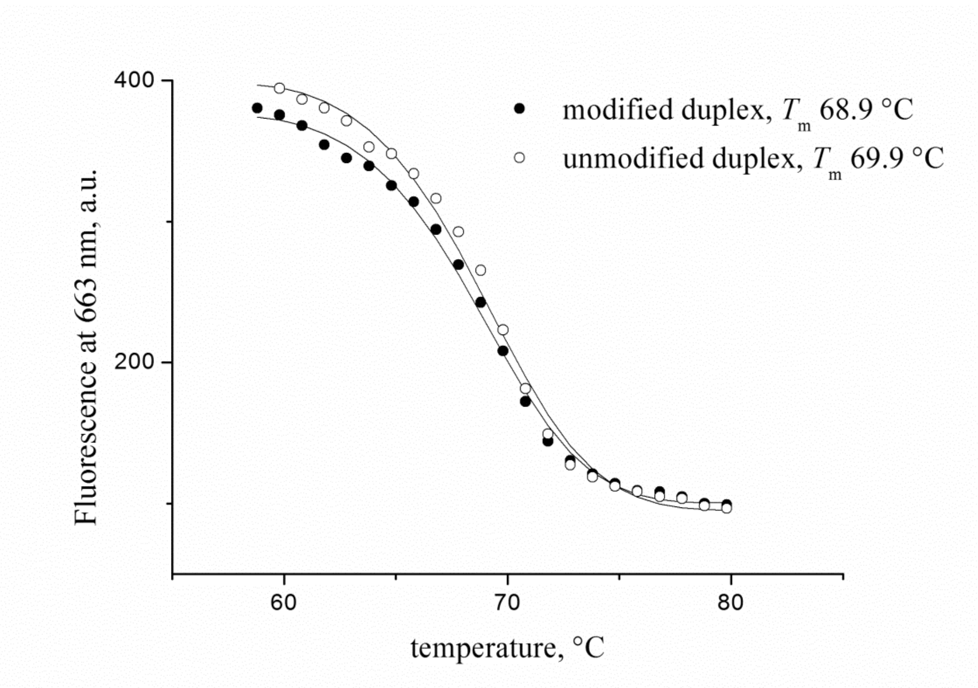

Figure S3. FRET melting profiles of full size DNA duplexes formed by the labelled strand M1 and native or modified primer strand.

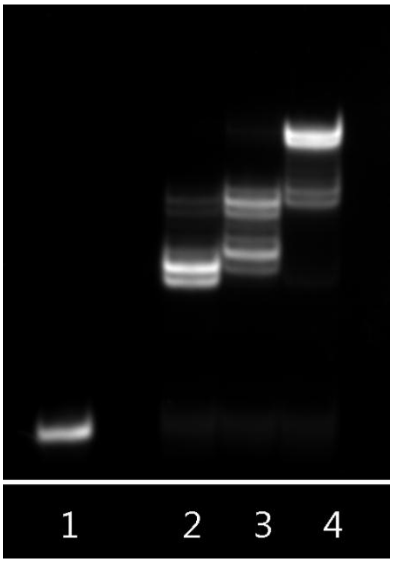

Figure S4. Electrophoretic separation of the labelled extension products formed in the PEX reaction with TTP using the template 5’-A_5-L-A_8TTGTCACTCAGACCAACTCCCT-NH_2 in the presence of Taq DNA polymerase. Lanes: (1) primer; (2) 5 min, 64 °C; (3) 60 min, 64 °C; and (4) 60 min, 72 °C.

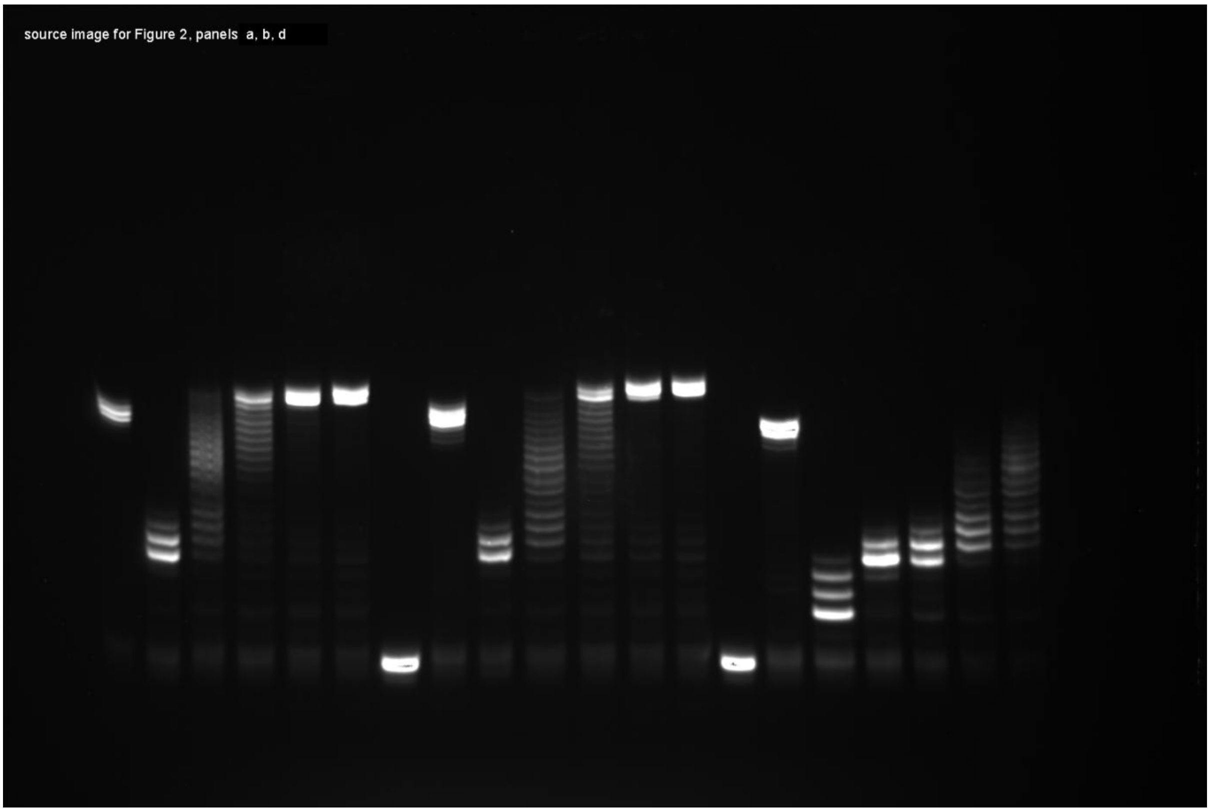
source image for Figure 2, panels a, b, d

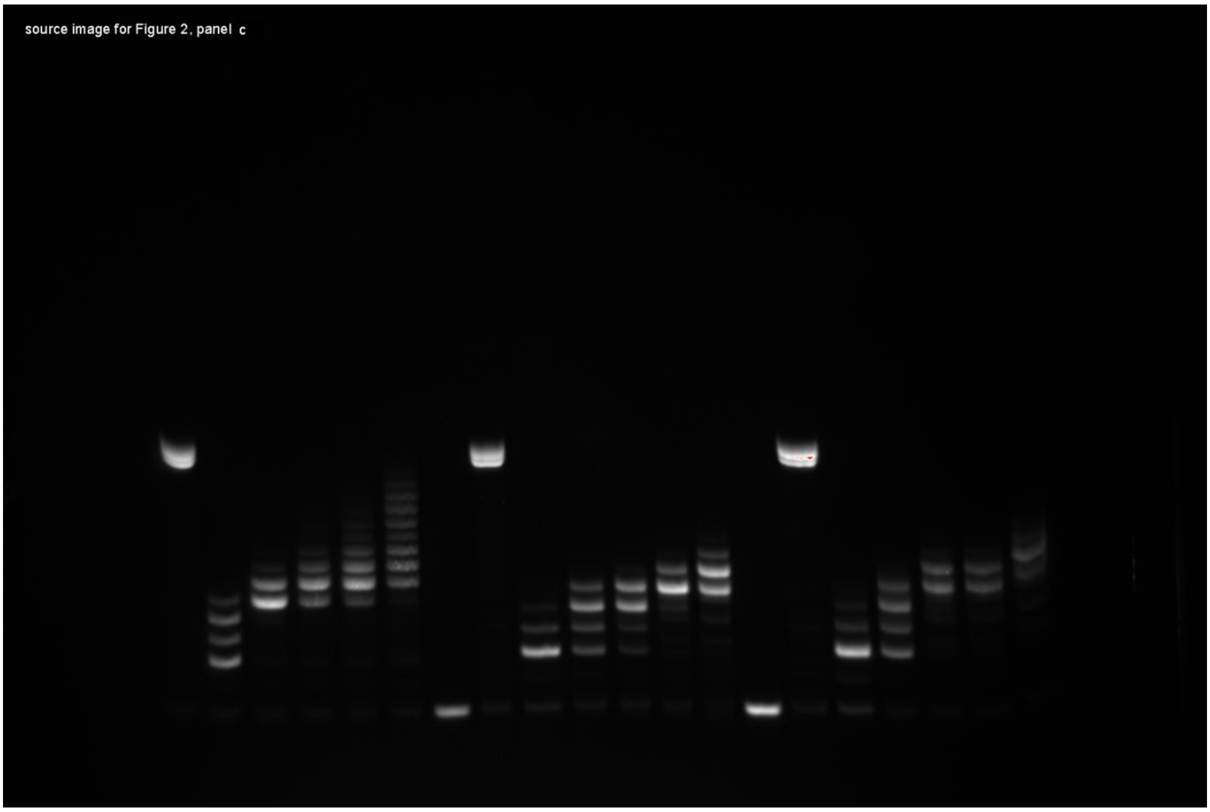
source image for Figure 2, panel c

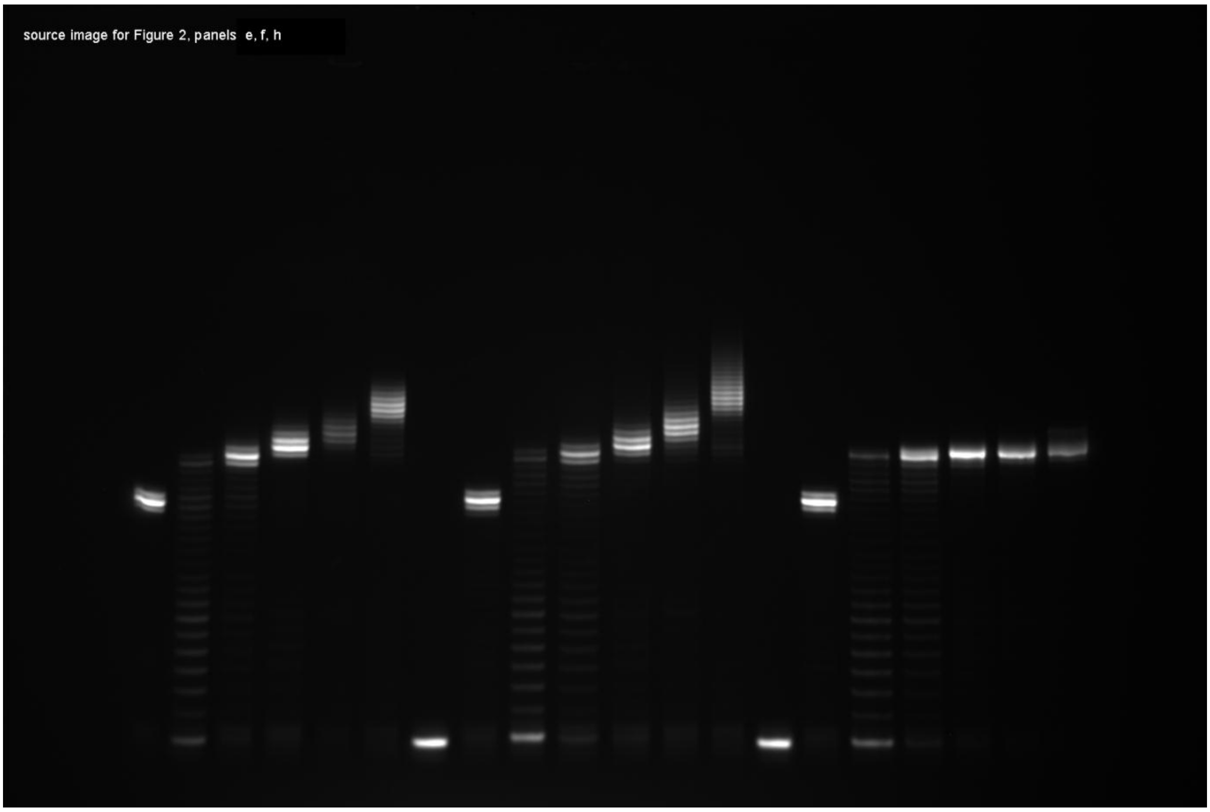
source image for Figure 2, panels e, f, h

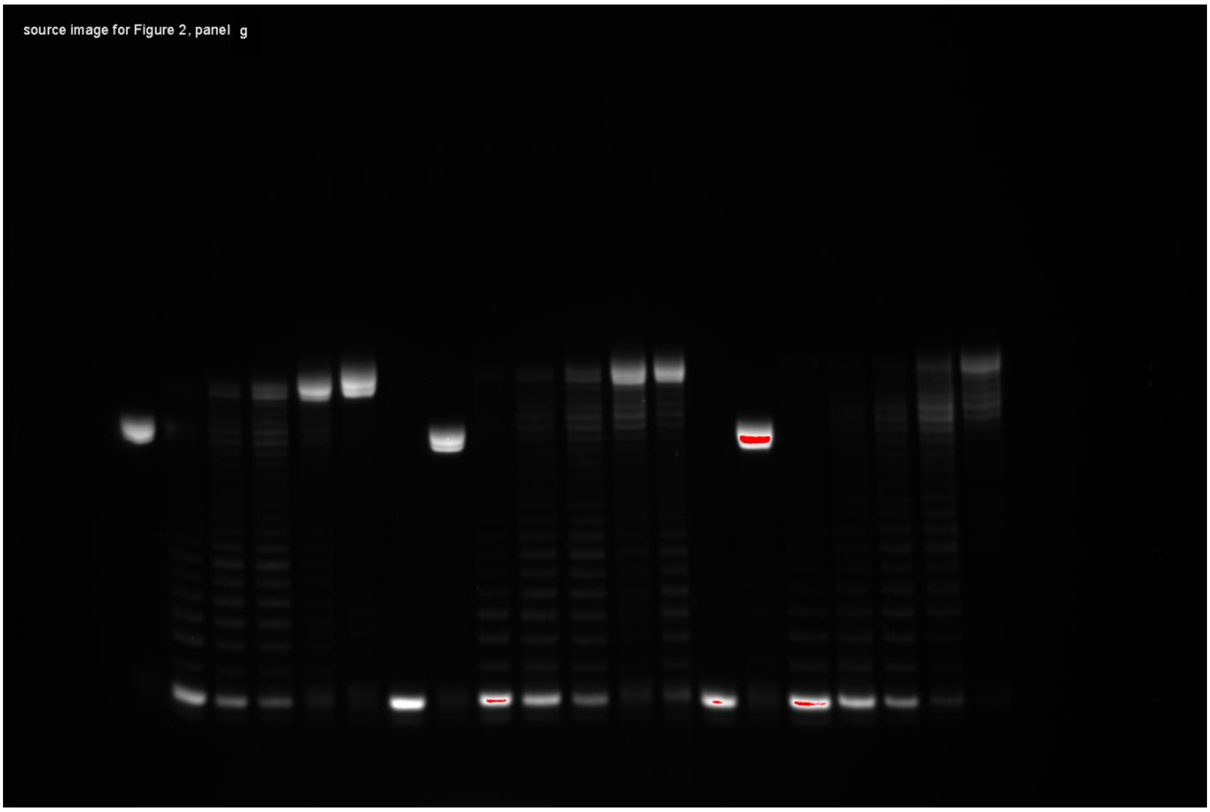
source image for Figure 2, panel g

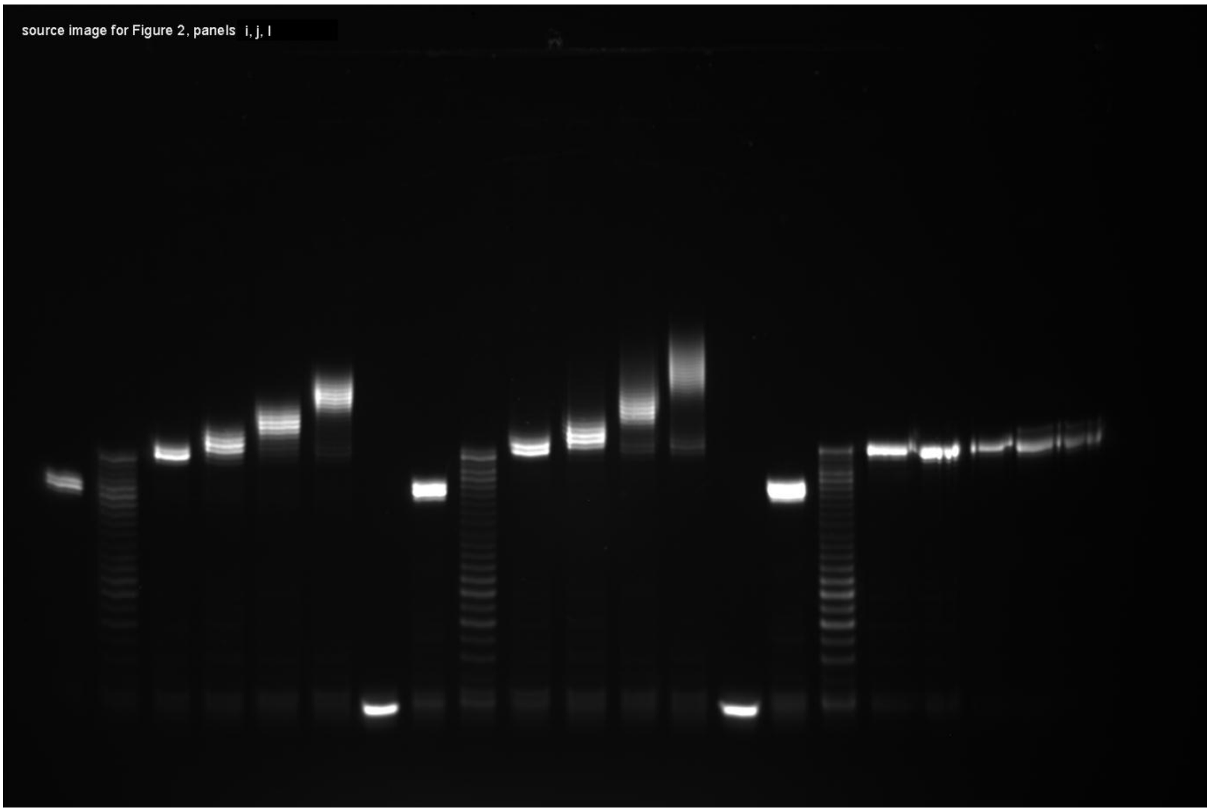
source image for Figure 2, panels i, j, l

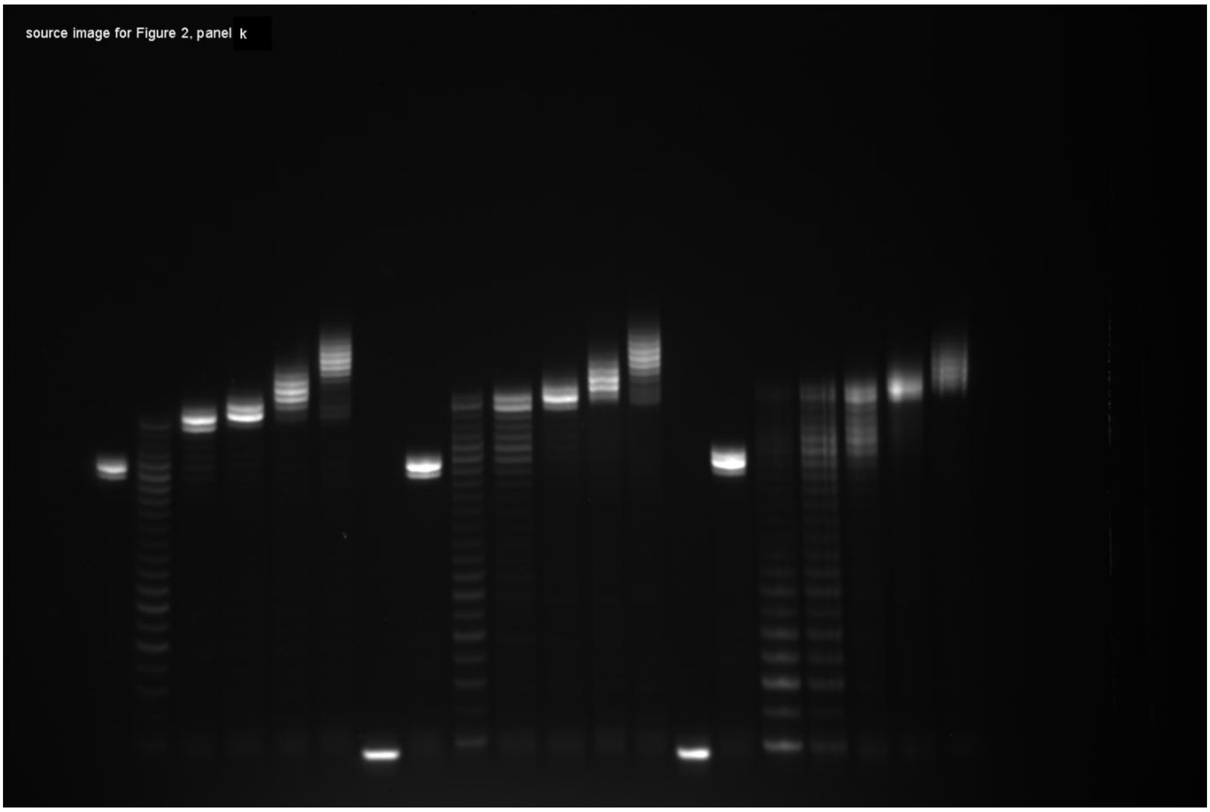
source image for Figure 2, panel k

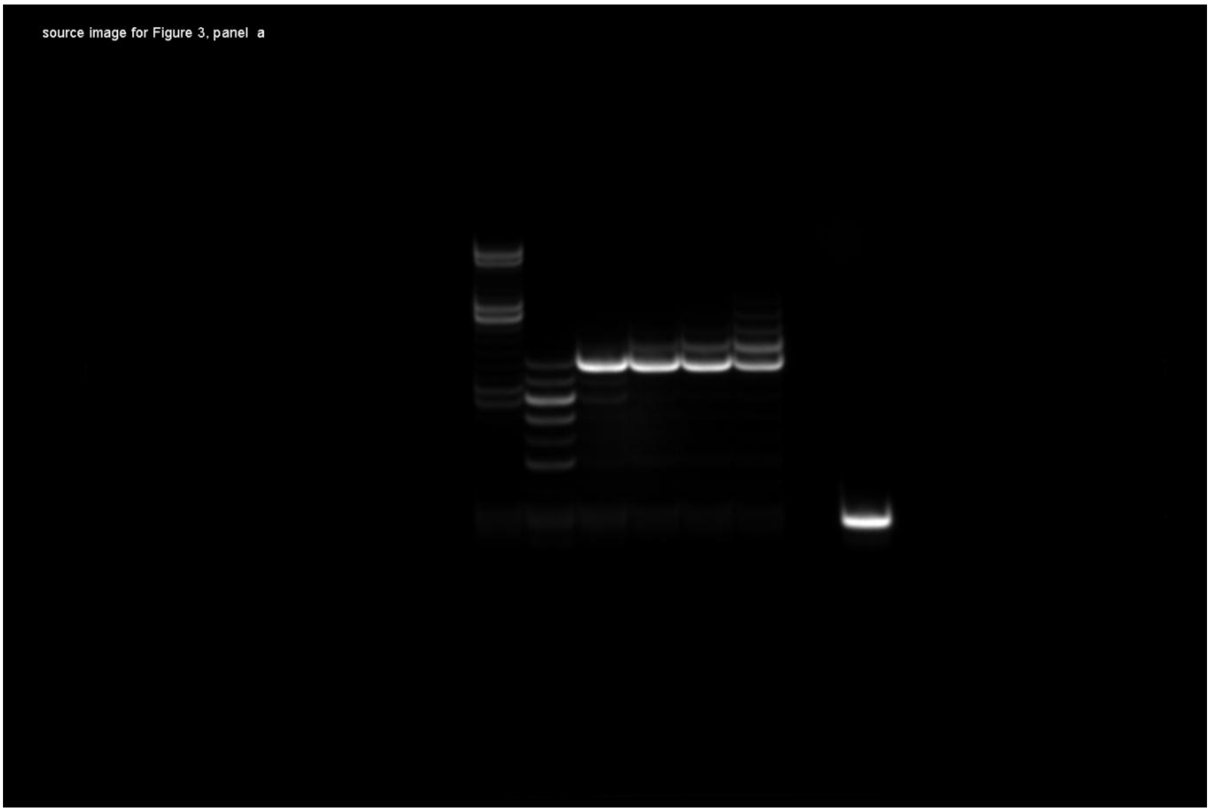
source image for Figure 3, panel a

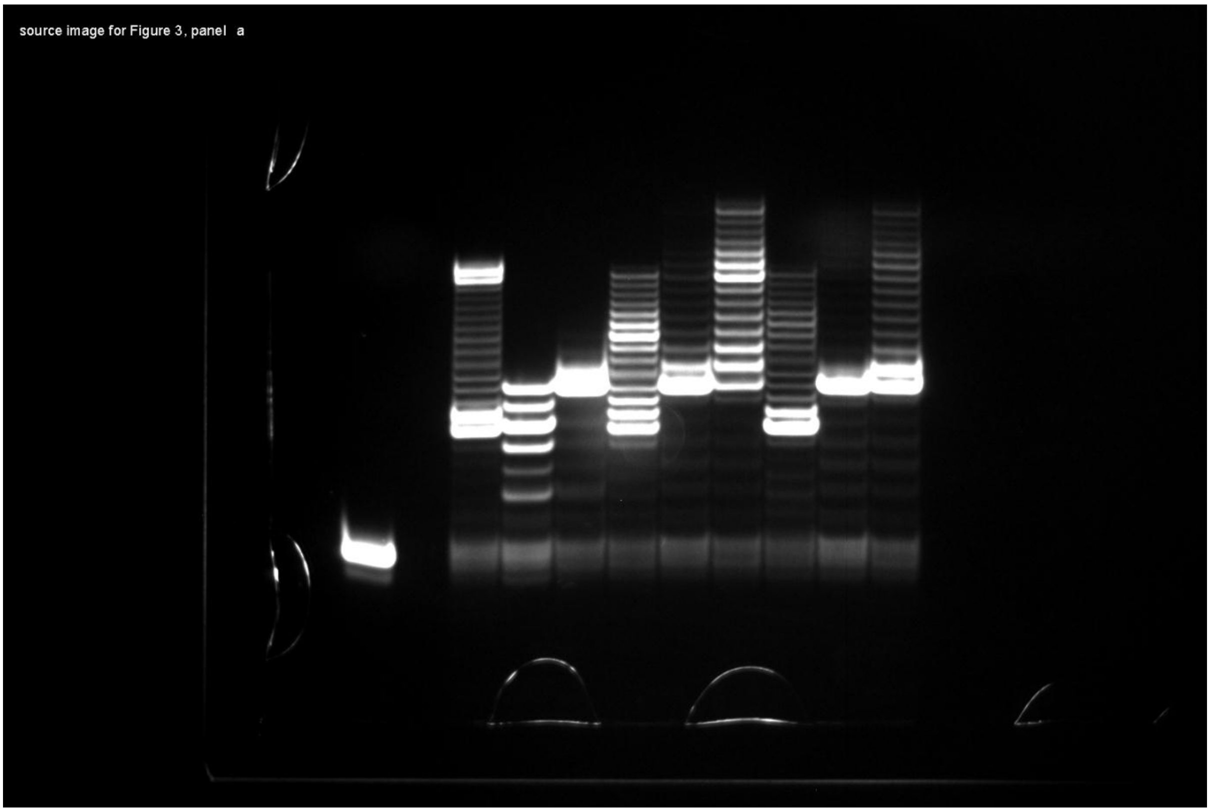
source image for Figure 3, panel a

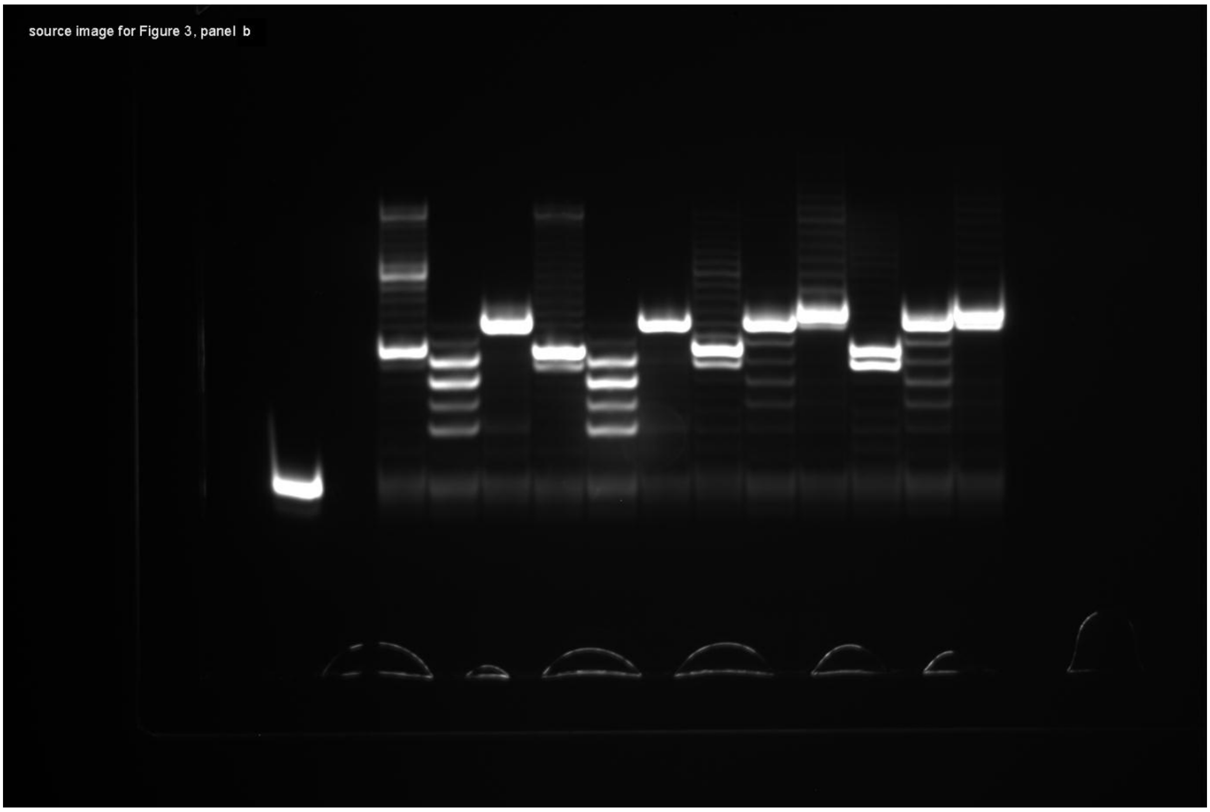
source image for Figure 3, panel b

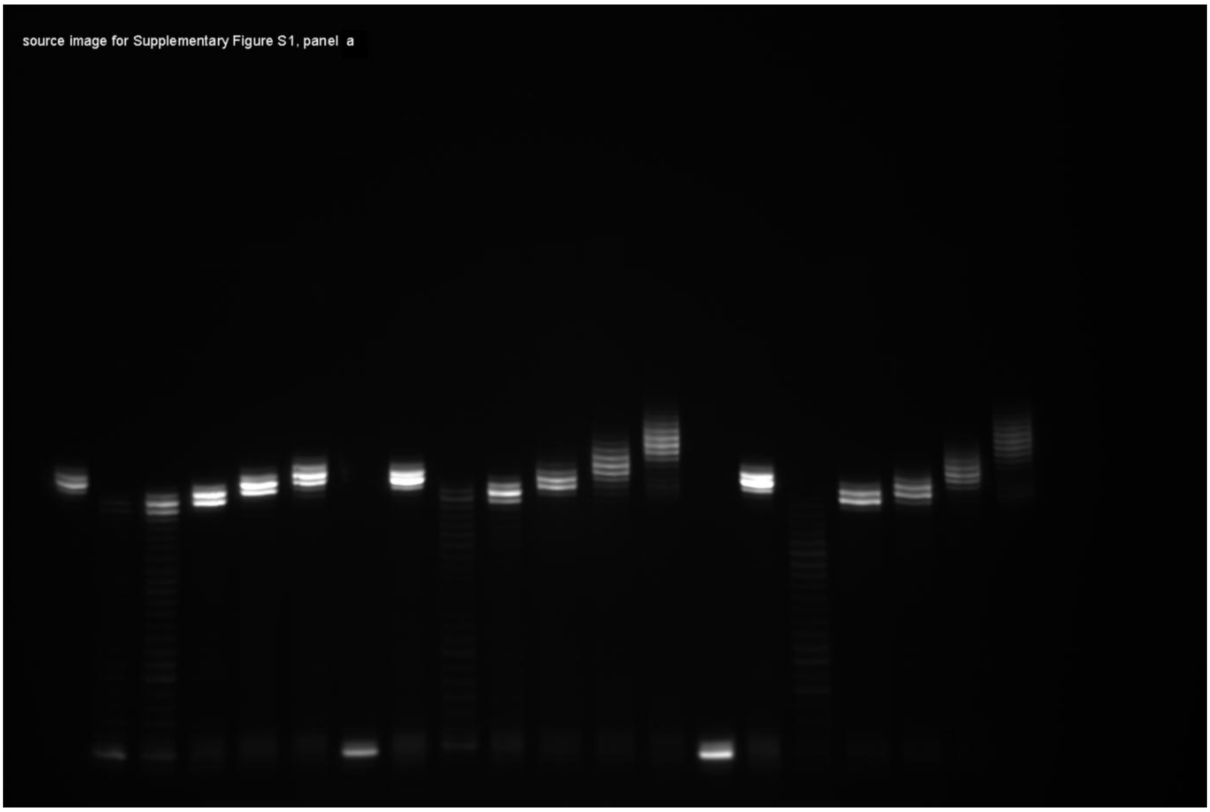
source image for Supplementary Figure S1, panel a

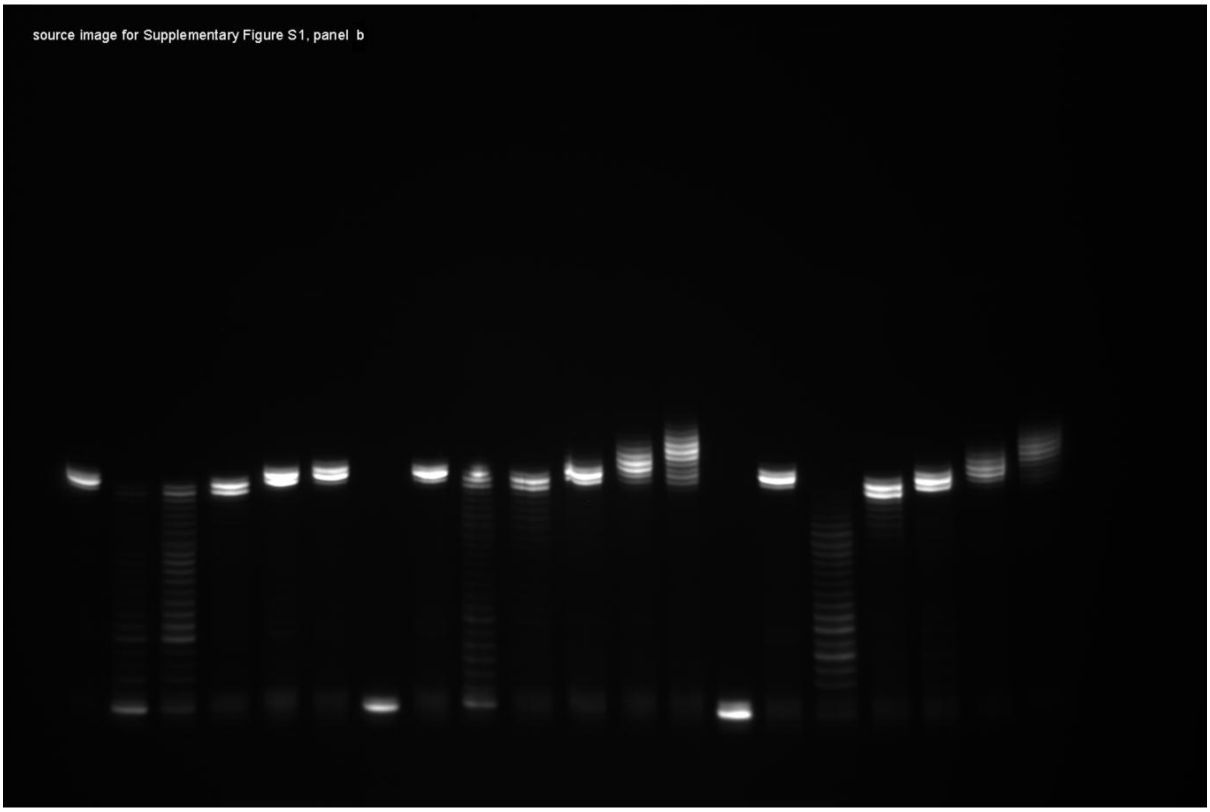
source image for Supplementary Figure S1, panel b

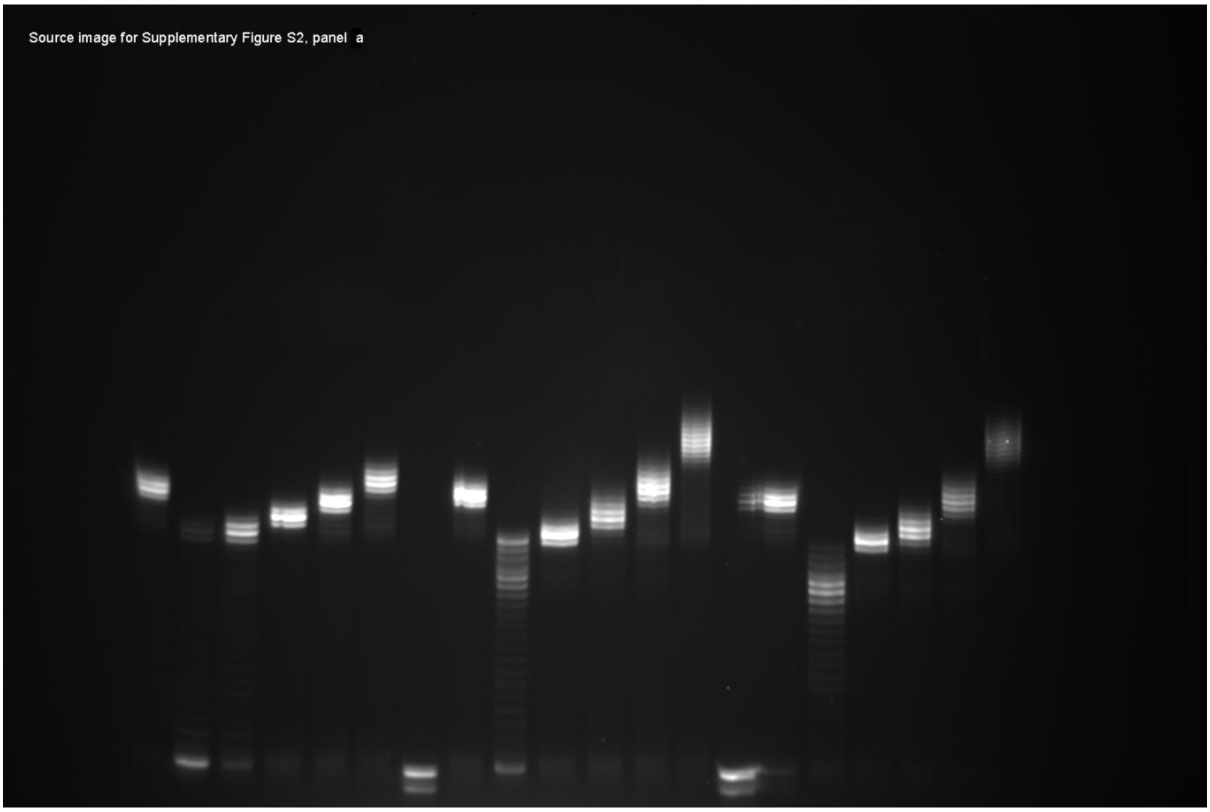
Source image for Supplementary Figure S2, panel a

Figure S5. Source gel images for Figures 2, 3, S1, S2 and S4.

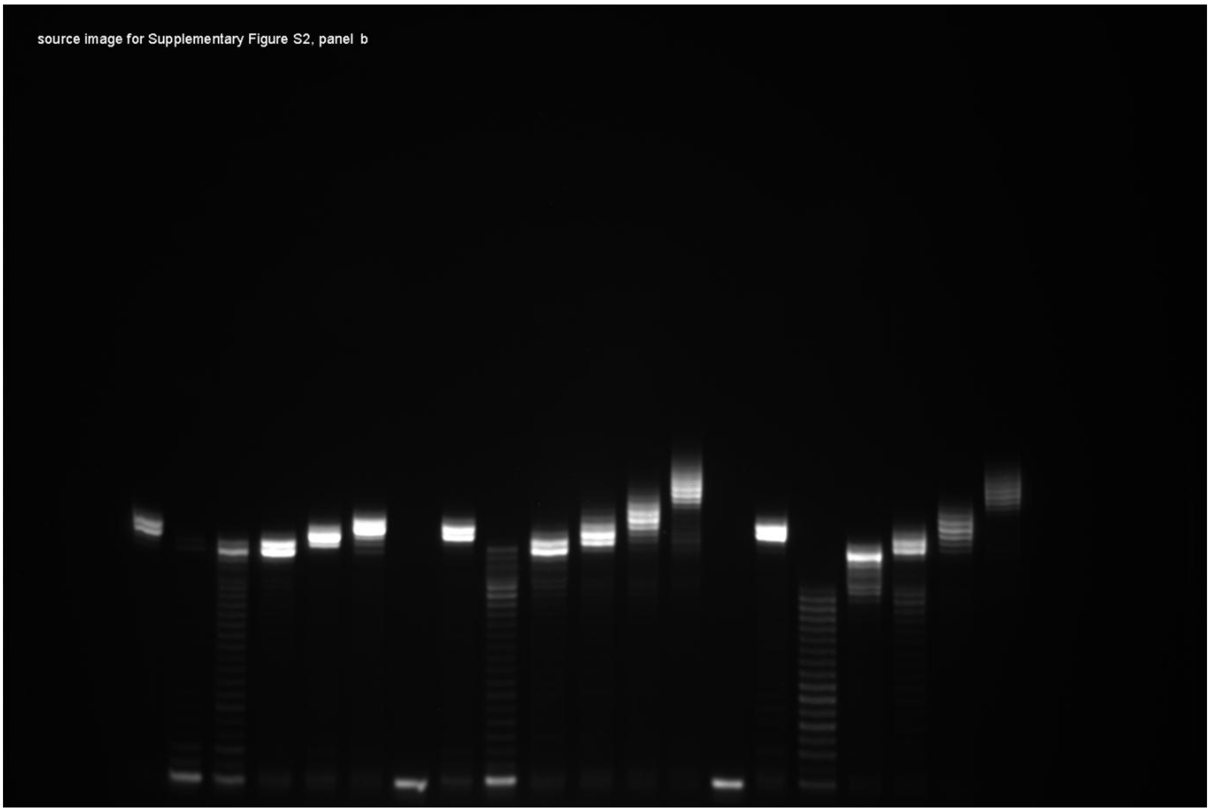

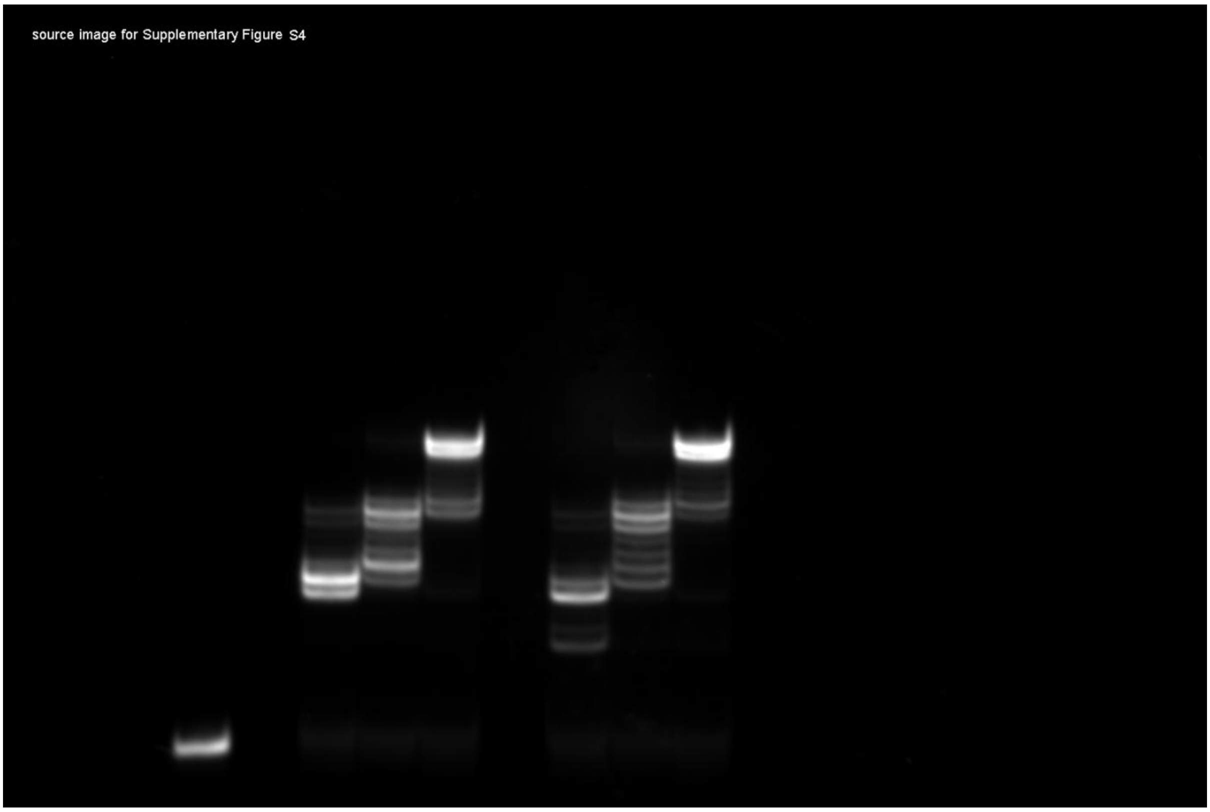

Figure S5. Source gel images for Figures 2, 3, S1, S2 and S4.
